# Growth Inhibitory Effects of Antimicrobial Natural Products against Cariogenic and Health-Associated Oral Bacterial Species

**DOI:** 10.3290/j.ohpd.a44307

**Published:** 2020-07-04

**Authors:** Nebu Philip, Shaneen Leishman, HMHN Bandara, Laurence Walsh

**Affiliations:** a Doctoral Candidate, School of Dentistry, The University of Queensland, Brisbane, Australia. Performed the experiments, data analysis and interpretation, drafted the manuscript.; b Postdoctoral Fellow, School of Dentistry, The University of Queensland, Brisbane, Australia. Experimental design, data analysis, manuscript revision.; c Postdoctoral Fellow, School of Dentistry, The University of Queensland, Brisbane, Australia. Experimental design, data analysis, manuscript revision.; d Professor, School of Dentistry, The University of Queensland, Brisbane, Australia. Conception and hypothesis, contributed substantially to discussion, critically revised and approved the manuscript.

**Keywords:** dental caries, natural products

## Abstract

**Purpose:**

This study investigated whether selected natural products could specifically target the growth of a caries-associated bacterial species (*Streptococcus mutans*) without affecting the viability of a health-associated oral commensal bacterial species (*Streptococcus sanguinis*).

**Materials and Methods:**

Agar diffusion assays were used to screen the natural products for bacterial-growth inhibitory effects and the diameters of the inhibitory zones for the two bacterial species compared. The minimum inhibitory concentrations (MIC) of the natural products that showed growth inhibitory effects were determined using the broth microdilution method.

**Results:**

Except for the berry extracts (cranberry, wild blueberry, and strawberry), all the other selected natural products (peppermint, ginger, cinnamon, rosemary, liquorice, xanthorrrhizol, tt-farnesol, guaijaverin, and macelignan) exhibited varying degrees of bacterial growth inhibition. The MIC values ranged from as low as 4 µg/ml for xanthorrrhizol to 1000 µg/ml for guaijaverin. All the growth inhibitory natural agents tested showed similar inhibition for both *S. mutans* and *S. sanguinis*.

**Conclusions:**

Although several natural products exerted significant antibacterial effects, none had selective inhibitory action on the growth of *S. mutans*.

Dental caries remains among the most widespread global oral health problems, affecting quality of life and imposing a costly burden on health services.^16^ The 2018 Australian Institute of Health and Welfare report highlighted that 42% of Australian children aged between 5 and 10 years experience caries, while 30% of the adults in the 25-40 years age bracket have untreated caries lesions.^1^ Moreover, the disease is not uniformly distributed, with many population groups, including indigenous children and the elderly, at increased risk of developing the disease.^10^

In addition to the established caries preventive methods, adjunctive measures commensurate to individual caries risk are often needed to control the disease in high-risk populations. With caries known to be a disease of microbiome dysbiosis, preventive strategies that ecologically modify the dental plaque biofilm are being increasingly recommended.^25^ Health-associated microbiomes can deliver small but relevant benefits over a prolonged period, and could be particularly useful for long-term caries control.^3,20^ With the aim of reducing levels of mutans streptococci (MS), a range of agents have been used, including biocides (chlorhexidine,^26^ triclosan,^4^ and cetylpyridinium chloride^13^) and antibiotics (vancomycin^7^). However, a concern with conventional antimicrobial agents is that they usually exert a broad-spectrum of antibacterial action, suppressing even health-associated oral microbial communities, and thus disrupting key health benefits of the resident oral microbiome.^27^ Furthermore, the side-effects associated with synthetic antimicrobials limit their acceptability and the duration for which they can be used. Once the chemotherapeutic intervention stops, susceptible tooth surfaces are often repopulated with the same disease-associated microbiome that was eliminated.

Plant-derived natural products with bacterial-growth inhibitory effects are an attractive alternative to traditional oral biocides for long-term caries prevention. Phytochemicals have been shown to reduce the development of dental plaque, influence bacterial adhesion, and reduce symptoms of oral diseases.^23^ For caries prevention, such natural agents should ideally supress the growth of MS without significantly inhibiting commensal health-associated plaque microflora. There is comparatively little information in the literature on whether any of the reported antimicrobial natural products can selectively inhibit the growth of cariogenic bacteria without affecting ‘healthy’ bacteria. Therefore, the aim of this study was to compare the growth inhibitory effects of a range of natural products on the caries-associated *Streptococcus mutans* and the health-associated *Streptococcus sanguinis* bacterial species.

## Materials and Methods

### Test Agents

The selection of test agents was based on a literature review that identified potential cariostatic natural products. High-quality supercritical CO_2_ extracts of peppermint, cinnamon, ginger, rosemary, liquorice, and xanthorrrhizol were sourced from Flavex Naturextrakte (Rehlingen, Germany). Water soluble molecular extracts of cranberry, wild blueberry, and strawberry were sourced from Diana Food (Champlain, Canada). In addition, phytochemicals found in bee-hive propolis (tt-farnesol) (Sigma-Aldrich; Sydney, Australia), and those isolated from guava leaves (guaijaverin) and nutmeg (macelignan) (Seebio Biotech; Shanghai, China) were also selected. Stock solutions of all the natural products were prepared, with the berry extracts dissolved in phosphate buffered saline (PBS), while the other test agents were suspended in 2% dimethyl sulfoxide (DMSO, Sigma-Aldrich; St Louis, MO, USA). Both PBS and the 2% DMSO were used as vehicle controls in the different experiments.

### Bacterial Strains

Bacterial cultures were obtained from the American Type Culture Collection (ATCC; Manassas, VA, USA). *S. mutans* ATCC 25175 and *S. sanguinis* ATCC 10556 were revived from freeze-dried vials and cultured on trypticase soy agar plates (Becton Dickinson, CA, USA) supplemented with 5% defibrinated sheep blood and incubated in a 5% CO_2_ atmosphere at 37°C for 72 h. The bacterial colonies were then subcultured in brain heart infusion (BHI, Merck; Darmstadt, Germany) and incubated overnight at 37°C. The resulting bacterial cultures were centrifuged at 4000 x g for 5 min, washed twice with PBS, and resuspended in BHI. The bacterial cell count in the culture medium was spectrophotometrically adjusted to approximately 2 x 10^8^ CFU/ml just before each assay.

### Agar Diffusion Assay

A standard agar diffusion assay was used for the initial screening of growth inhibitory effects against *S. mutans* and *S. sanguinis*.^9^ Briefly, five wells of 5 mm diameter were punched into pre-prepared Mueller-Hilton agar petri dishes (MH agar, ThermoFisher Scientific; Waltham, MA, USA). The wells were placed 30 mm apart and 20 mm from the outer edge of the petri dish. Each well was loaded with a fine spiral of sterile filter paper (90 mm x 4 mm). Aliquots (50 µl) of the respective test agent solutions/controls were then carefully pipetted onto the filter paper. Each plate had three test agents, plus chlorhexidine (CHX) (0.2% w/v) as a positive control, and the appropriate vehicle control. For each plate, 5 ml aliquots of the standardized bacterial suspension was added to 5 ml of melted MH agar at 45°C, mixed thoroughly and poured evenly over the surface of the agar plates containing the wells loaded with test agents/controls. After incubation at 37°C for 24 h, the plates were assessed for bacterial growth or inhibition. The zones of inhibition were measured directly at their minimum diameter. All experiments were performed in triplicate on three independent occasions.

### Broth Microdilution Assay

The minimum inhibitory concentration (MIC) of the test agents was determined using the broth microdilution method as specified by the Clinical and Laboratory Standards Institute.^6^ Briefly, two-fold serial dilutions of the test agent stock solutions were prepared and pipetted into separate wells of 96-well microtiter plates (Costar 3596, Corning; Corning, NY, USA). The wells were then inoculated with the bacterial suspensions such that the final bacterial concentration in each well was approximately 5 x 10^5^ CFU/ml. Triplicate samples were prepared for each concentration of the agent being tested. Appropriate solvent control, growth control, and sterility control wells were also maintained in each microtiter plate. After incubation for 24 h at 37°C, the optical density (OD) was determined at 600 nm wavelength in a microplate spectrophotometer (Tecan Infinite 200 Pro; Grodig, Austria) after correcting for the background absorbance of the test agent solutions. The MIC of each test agent against *S. mutans* and *S. sanguinis* was calculated from the adjusted OD values obtained from three independent experiments.

### Statistical Analysis

After data sets were assessed for normality, an independent sample t-test was used to evaluate differences in the zones of growth inhibition between *S. mutans* and *S. sanguinis* bacterial species for each test agent. The level of significance was set at 5%. Statistical software SPSS version 24 (IBM; Armonk, NY, USA) was used to perform the analysis.

## Results

Except for the three berry extracts, all the other natural products inhibited the growth of both *S. mutans* and *S. sanguinis* in the agar diffusion assay. The highest inhibition of bacterial growth was seen for xanthorrrhizol, followed by macelignan and tt-farnesol (Table 1). The diameters of the zones of inhibition for *S. mutans* were not significantly different from that for *S. sanguinis* for any of the growth inhibitory natural products tested (p > 0.05).

**Table 1:** Growth inhibitory effects of natural products against *Streptococcus mutans* and *Streptococcus sanguinis*

Test agent (concentration)	Zone of inhibition diameter (mm)		p-value (SM vs SS)
	*Streptococcus mutans* (SM)	*Streptococcus sanguinis* (SS)	
Xanthorrrhizol (200 µg/ml)	13.2 ± 0.44	13.1 ± 0.58	NS
Macelignan (200 µg/ml)	11.8 ± 0.67	12.2 ± 1.13	NS
tt-Farnesol (200 µg/ml)	11.6 ± 0.45	11.3 ± 0.33	NS
Liquorice (1 mg/ml)	10.8 ± 0.86	11.0 ± 0.79	NS
Cinnamon (1 mg/ml)	9.7 ± 0.98	9.9 ± 0.54	NS
Peppermint (1 mg/ml)	9.2 ± 0.44	9.4 ± 0.48	NS
Ginger (1 mg/ml)	8.4 ± 1.26	8.3 ± 1.30	NS
Rosemary (1 mg/ml)	8.1 ± 0.66	7.8 ± 0.84	NS
Guaijaverin (1 mg/ml)	7.2 ± 0.72	7.2 ± 0.55	NS
Cranberry (16 mg/ml)	0	0	–
Wild blueberry (16 mg/ml)	0	0	–
Strawberry (16 mg/ml)	0	0	–
Vehicle control (2% DMSO/PBS)	0	0	–
Positive control (0.2% CHX)	17.7 ± 0.89	18.0 ± 0.98	NS

Diameter of inhibitory zones (mean ± SD) from three independent triplicate experiments (n = 9). DMSO: dimethyl sulfoxide; PBS: phosphate buffered saline; CHX: chlorhexidine. NS: no significant differences between *S. mutans* and *S. sanguinis* inhibition using the independent sample t-test (p > 0.05).

The MICs of the natural products that exhibited bacterial growth inhibition is presented in Table 2. The MIC values obtained from the microdilution assays followed the bacterial growth inhibitory pattern seen in the agar diffusion assay, with xanthorrrhizol showing the lowest MIC (4 µg/ml) against both *S. mutans* and *S. sanguinis* among all the natural products tested.

**Table 2:** MIC of natural products with bacterial growth inhibitory effects (µg/ml)

Test agent	*Streptococcus mutans*	*Streptococcus sanguinis*
Xanthorrrhizol	4	4-8
Macelignan	8	4
tt-Farnesol	8	8
Liquorice	80	100
Cinnamon	340	175
Peppermint	375	340
Ginger	275	475
Rosemary	800	800
Guaijaverin	1000	1000
CHX (control)	1	1

MIC: minimum inhibitory concentration calculated from three independent triplicate experiments (n = 9). CHX: chlorhexidine.

## Discussion

The results of this study provide insights into the growth inhibitory effects of selected natural products against *S. mutans* and *S. sanguinis* bacteria. *S. mutans* is one of the primary bacterial culprits responsible for caries pathogenesis due to its acidogenic, aciduric, and glucan synthesis properties. On the other hand, *S. sanguinis* is able to use its arginine deaminase system to neutralize acids and slow caries lesion progression. These bacterial species were thus chosen as being representative of those associated with caries and health, respectively.

None of the fruit berry extracts (cranberry, blueberry, and strawberry) demonstrated bacterial growth inhibitory effects against either *S. mutans* or *S. sanguinis* despite their high polyphenol content. This suggests that the bioactive phytochemicals present in these berry extracts may lack bactericidal effects at the concentrations tested. In contrast, all the other selected natural products inhibited the growth of both *S. mutans* and *S. sanguinis* with varying degrees of potency. Xanthorrrhizol, the bioactive compound in Javanese turmeric (*Curcuma xanthorrhizha*) showed the highest bacterial growth inhibition, followed closely by macelignan from nutmeg (Myristica fragrans), and tt-farnesol, a sesquiterpene isolated from bee-hive propolis. The MICs of the tested natural agents ranged from 4 µg/ml for xanthorrrhizol, up to 1000 µg/ml for the guaijaverin compound found in guava (*Psidium guajava*).

There were no significant differences observed between *S. mutans* and *S. sanguinis* growth inhibition for any of the natural products tested (p > 0.05). Considering oral streptococci have common structural properties,^2,12^ it will be challenging to find an agent that can selectively suppresses the growth of cariogenic MS without also affecting health-associated oral streptococci such as *S. sanguinis* or *Streptococcus mitis/oralis*. It would be worthwhile to investigate whether non-streptococcal health-associated plaque microflora (e.g. *Neisseria flava* or *Corynebacterium durum*), can remain relatively unaffected by the antimicrobial effects of these natural products. While other studies have shown that plant-derived essential oils present in mouthrinses can reduce MS levels and total plaque,^11,21,22^ their ability to discriminate between ‘harmful’ and ‘healthy’ microflora requires further study.

The MICs of xanthorrrhizol and macelignan against *S. mutans* (4 µg/ml and 8 µg/ml respectively) found in this study were consistent with previous reports on these phytochemicals.^5,14^ While MIC values of these phytochemicals may seem only slightly higher than that of CHX (1 µg/ml), it is important to consider the molecular weight of these compounds when comparing the antimicrobial potency of various agents. For example, the 4 µg/ml MIC of xanthorrrhizol corresponds to 18.3 µmol/ml based on its molecular weight (MW) of 218.3 g/mol, while the similar MIC of CHX (MW 897.8 g/mol) is only 1.1 µmol/ml. Evidently, CHX is at least 16-fold more potent than xanthorrrhizol against these bacterial species. However, the demonstrated MICs of xanthorrrhizol (18.3 µmol/ml) and macelignan (24.4 µmol/ml) were much lower than the reported MICs of essential oils like menthol (3200 µmol/ml) and thymol (3329 µmol/ml) that are commonly used in mouthrinses for antibacterial effects. This suggests their excellent potential for incorporation into oral care products as alternatives to the currently used antibacterial essential oils in conditions where broad antimicrobial action is required. For the other natural extracts tested in this study, identification and isolation of their bioactive antimicrobial compounds could further improve their antibacterial effects.

Recent reports have underlined the symbiotic benefits a healthy oral microbiome affords the host.^8,17^ This has led to clinical recommendations for adopting ecological strategies as part of modern caries management.^24^ Clearly, long-term control of caries risk should be based on successful “stewardship” of the plaque biofilm, rather than simply focussing on eliminating it. This will allow health-associated microbial communities to dominate the dental plaque biofilm, and thereby lower its virulence potential. From an ecological perspective, it may thus be more beneficial to use natural products that disrupt cariogenic virulence properties (e.g. acidogenicity or glucan synthesis), rather than those that broadly affect bacterial viability and growth.^15^ For example, specific phytochemicals were able to modulate cariogenic virulence and inhibit dental caries in vivo despite lacking significant biocidal activity.^18,19^

## Conclusion

While this screening study could not identify antimicrobial natural products that specifically targeted the growth of cariogenic bacteria, further studies using natural agents with potential for inhibiting plaque virulence properties are currently underway. The vast therapeutic potential of phytodentistry in lowering caries-risk and preventing oral diseases remains to be fully exploited.
